# Supplementary material for: Silencing the CsSnRK2.11 Gene Decreases Drought Tolerance of Cucumis sativus L
Source: Int J Mol Sci. 2023 Oct 30;24(21):15761. doi: 10.3390/ijms242115761 (PMC10649623; doi:10.3390/ijms242115761)
Supplement: Supplementary file 1 [file ijms-24-15761-s001.zip › Supplementary materials S1.pdf]

Table S1. Primers used for colony PCR validation and Silencing efficiency qRT-PCR analysis

Primer name	Primer sequence(5'-3')
SnRK2.11-F	TGAGTAAGGTTACCGAATTCCACTTCTAGATGGAAGCTCAGCC
SnRK2.11-R	GGACATGCCCGGGCCTCGAGTTCTTACATAGTGTGGTATTGA
qRT-PCR-F	CAGACCGAAGAAGTTGTTGCCATG
qRT-PCR-R	ACCAAAGCCGCGTGGGTTTTG
actin-F	GCCCTCCCTCATGCCATTCT
actin-R	TCGGCAGTGGTGGTGAACAT
