# Supplementary material for: Silencing the CsSnRK2.11 Gene Decreases Drought Tolerance of Cucumis sativus L
Source: Int J Mol Sci. 2023 Oct 30;24(21):15761. doi: 10.3390/ijms242115761 (PMC10649623; doi:10.3390/ijms242115761)
Supplement: Supplementary file 1 [file ijms-24-15761-s001.zip › Supplementary materials S2.pdf]

Table S2. Primers for qRT PCR of differentially expressed genes in Transcriptome data

Gene Name	Forward Primer Sequence (5'-3')	Reverse Primer Sequence (5'-3')
CsaV3_3G018770	CTAAATACTTTGGCGGTGGT	GAACGAACGCTTCTTGATGT
CsaV3_3G018760	CTTCTTGCTCGGTGTATTGTG	CCTGAACCTCTGGGAATGTC
CsaV3_1G042490	ACTATACCCACCAACACCCA	CATTCACAATTTCACGTCCC
CsaV3_3G018750	TTTCCAGCACAAAGCCAGAG	AGGCCAAGAATACGACTTCCAT
CsaV3_7G002170	CCCCAGGGTCAAACTCAAGG	GATGCCAAAGAATCCAATAAGGTA
CsaV3_6G048960	GAAGGTCGGGAAGTTTAGAG	GGAATGTTTAGGGAAGAAGAG
CsaV3_2G012870	GAATGACTCCGACGAAATGC	AATGTCCCGAGCCAAACCCT
CsaV3_7G028990	CAAGCCGCCTTCCTTCGTAA	TCCCAATTCGCGTCGTATCT
CsaV3_1G007040	GCCTGGCTACCAACAAGACT	ATTCCCTCCACCTGATACGC
CsaV3_4G032550	GACTTGTTGGGTGGGTTGAT	ACCAGAGGGAGGGTGTTTGC
Actin	GCCCTCCCTCATGCCATTCT	TCGGCAGTGGTGGTGAACAT
